# Multidomain evaluation and data-driven approaches to predict recurrent neck pain (END-RNP): a study protocol for a multicentre prospective longitudinal cohort study

**DOI:** 10.1136/bmjopen-2025-112430

**Published:** 2026-02-27

**Authors:** Valter Devecchi, Bernard Liew, Jonathan Price, Kym Snell, Richard Riley, Deborah Falla

**Affiliations:** 1 Centre of Precision Rehabilitation for Spinal Pain (CPR Spine), School of Sport, Exercise and Rehabilitation Sciences, University of Birmingham, Birmingham, UK; 2 School of Sport, Rehabilitation and Exercise Sciences, University of Essex, Essex, UK; 3 Musculoskeletal Physiotherapy Services, Birmingham Community Healthcare NHS Foundation Trust, Birmingham, UK; 4 School of Sport, Exercise and Rehabilitation Sciences, University of Birmingham, Birmingham, UK; 5 Department of Applied Health Sciences, University of Birmingham, Birmingham, UK; 6 NIHR Birmingham Biomedical Research Centre, Birmingham, UK

**Keywords:** REHABILITATION MEDICINE, Musculoskeletal disorders, Chronic Pain, Prognosis

## Abstract

**Introduction:**

Neck pain (NP) is a leading cause of disability worldwide and affects more than 200 million people. Incidence and associated economic burden are constantly increasing, and little is known about the factors that promote new and more severe episodes in those individuals with recurrent NP. Current evidence supports that changes in physical, psychological and social factors persist between NP episodes, and these changes might contribute to the development of new episodes. The End Recurrent Neck Pain (END-RNP) study aims to use physical, psychological and social factors tested while in symptom remission to predict, within a 12-month period, the frequency and severity of new NP episodes.

**Methods and analysis:**

The END-RNP study is a multicentre, prospective cohort study conducted from March 2025 to February 2028 at the University of Birmingham and the University of Essex (UK). 300 adults reporting two or more NP episodes in the previous year will be recruited from September 2025 to form the recurrent NP cohort, and 48 adults without a history of NP will provide normative data. Laboratory testing will be conducted for all participants when pain-free by assessing cervical kinematics and proprioception, neck-muscle strength, endurance and activation, pain processing, psychological and social factors. All recurrent NP participants will complete online questionnaires every 2 weeks for 12 months, recording days with NP, pain intensity/interference, healthcare use and other behavioural and environmental factors. Participants in the recurrent NP cohort who experience an acute NP episode during the 12-month follow-up will repeat the laboratory assessment. To develop the prediction models, candidate predictors will be the baseline measurements of any feature that shows either cross-sectional differences between recurrent NP and control groups or within-subject changes between the pain-free baseline and a pain episode. From the identified candidate predictors, two multivariable models will be developed using penalised regression, with (i) number of days with NP (linear regression) and (ii) NP severity (ordinal regression) as their respective dependent variables. Internal validation will use bootstrap resampling to estimate optimism-adjusted performance (R^2^, C-statistic and calibration slope), prediction instability and uncertainty, and clinical utility. The models from the END-RNP study will provide clinical prediction tools to help identify those at high risk of frequent and severe NP episodes and to inform the personalised prevention of recurrent NP.

**Ethics and dissemination:**

The END-RNP study was approved by the Ethics Committee at the University of Birmingham (ERN_4005-Aug2025) and by the University of Essex (ETH2526-0098) on 2 September 2025, prior to the recruitment of the first participant. The findings will be presented at national and international conferences and submitted for publication in peer-reviewed journals.

**STRENGTH AND LIMITATIONS OF THIS STUDY**

- This study will adopt a comprehensive biopsychosocial approach, including physical, psychological and social factors as potential predictors of future neck pain episodes within a longitudinal design.
- This study will be powered for the development of clinical prediction models and will use robust statistical methods for development, predictor selection and internal validation in accordance with established guidelines.
- The extensive and frequent follow-up will enable us to capture the temporal dynamics and episodic nature of recurrent neck pain.
- The developed models will be based on data collected in the UK. Further validation in new samples will be required to examine generalisability and transportability of the findings.
- All data will be collected within a laboratory setting; therefore, the generalisability of the findings and the feasibility of applying them in routine clinical settings will need to be investigated.

## Introduction

 Neck pain (NP) remains one of the leading causes of disability worldwide, resulting in financial burden and poor quality of life.^1 2^ In the UK, NP is second only to low back pain across the musculoskeletal causes of disability.^3^ While the total number of cases has risen with population growth and ageing, the global age-standardised incidence rate has also increased significantly since the early 2010s,^4^ with data from 2020 showing that more than 203 million people suffered from NP.^5^ This figure is expected to grow by more than 30% over the next three decades.^5^ Of those who experience an episode of NP, between 50 and 85% will report NP 1–5 years later,^6^ with over 20% of people developing recurrent pain episodes.^7^ Despite being a research priority,^8^ the factors contributing to the development of new episodes of NP remain poorly understood.^8^ Understanding these factors would allow targeted interventions and preventative measures.

People with NP present with impairments in physical, psychological and social function that may predispose them to ongoing pain or recurrence, as described by theories linking motor adaptations and psychosocial factors to pain.^9 10^ Clinical studies and systematic reviews have reported restricted range and speed of neck movement,^11 12^ poor quality of neck movement,^13 14^ altered muscle behaviour and sensorimotor control deficits^15 16^ in people with chronic or recurrent NP. Importantly, in individuals with recurrent NP, the resolution of pain symptoms does not necessarily correspond to a recovery of physical function; sensorimotor impairments similar to those observed in chronic NP often persist during symptom remission,^17^ potentially acting as a predisposing factor for future NP episodes. Lower pain pressure thresholds and altered conditioned pain modulation identified in people with NP also suggest impairment of central pain-modulating systems.^18 19^ The potential contributors to new NP episodes are not limited to physical factors, as psychological variables such as anxiety, stress, depression and low self-efficacy, along with social factors, can further modulate the pain experience.^20^,^23^ For example, social support, job satisfaction and other work-related factors have been associated with the development of NP.^24^ Given this multidimensional interplay, it is unlikely that any single factor explains NP recurrence; instead, complex interactions across domains could result in new NP episodes. Indeed, previous studies demonstrated how physical and psychological factors are highly interrelated in people with chronic and recurrent NP.^25 26^

Advancing the understanding of NP recurrence requires methodological approaches that acknowledge the multidimensional nature of NP, encompassing physical, psychological and social factors, and which employ methodologies capable of managing such complexity while monitoring NP progression over time. Although previous research has examined multiple domains within a biopsychosocial framework,^27^ individual and contextual physiological responses to pain have received less attention.^28^ Previous studies have predominantly focused on patient-reported outcomes, exploring what patients perceive and how they relate to their health condition, with limited attention to physical impairments associated with NP.^27 28^ In a recent systematic review, we found only six studies investigating predictors of recurrent NP episodes, with low quality of evidence and contrasting findings across studies for physical factors, and some limited evidence supporting the predictive role of psychological distress.^29^ However, a limitation of previous studies was the lack of a clear distinction between recurrent and chronic NP. While chronic NP involves persistent symptoms, recurrent NP is characterised by distinct episodes of pain separated by symptom-free remission periods. This makes it challenging to distinguish between the two if outcomes are evaluated at a single time point, overlooking the different symptom trajectories of recurrent versus chronic NP.^30^ Additionally, previous studies have typically assessed participants at baseline while in pain. Testing during an acute painful episode can introduce significant heterogeneity, as the presence of pain alters physical function and psychological states,^10 31 32^ potentially masking the role of relevant prognostic factors. Therefore, recruiting and testing individuals with recurrent NP during a pain-free remission period provides a more homogeneous sample. This allows for the identification of predictors of recurrence without the confounding influence of current pain. Evidence from patients with whiplash-associated disorders tested during a pain remission period suggests that physical impairments may predict future NP episodes.^33^ However, whiplash-associated disorders and idiopathic NP differ in aetiology and have been shown to present distinct physical and sensorimotor profiles.^34^,^36^ As such, findings from people with whiplash-associated disorders may not fully generalise to non-traumatic neck pain, highlighting the need to directly investigate prognostic physical factors in individuals with recurrent idiopathic NP. A comprehensive, multidimensional approach is therefore essential to address specifically the complexity of idiopathic NP and identify predictors of new NP episodes, while recognising the fluctuating and recurrent nature of NP, characterised by periods of symptom exacerbation and remission.^37^

### Aims and objectives

The primary aim of this study will be to develop and internally validate clinical prediction models for use in individuals with recurrent idiopathic NP to predict, within 12 months, the frequency and severity of new NP episodes using physical, psychological and social factors. This will be accomplished through two main objectives:

*O.1 Identify potential candidate predictors*. We will first identify potential candidate predictors by examining how neuromuscular control, neck function, pain processing, psychological and social features in individuals with a history of recurrent NP episodes differ from those in a control group without a history of NP, and how these components are affected during an acute new NP episode. By identifying clinically relevant features that change due to pain – whether they differ in people with a history of recurrent NP during remission compared with a control group, or whether they worsen during a new pain episode – this will aid in selecting potential candidate predictors. We hypothesise that some potential predictors of future NP episodes are those influenced by pain, either because they remain altered during pain remission when compared with a control group without history of NP, worsen during a new NP episode or both. In addition, the selection of candidate predictors will be informed by evidence from recent systematic reviews on prognostic factors for recurrent or persistent NP.^28 29^

*O.2 Prediction model development and internal validation*. Based on findings from the first objective, we will use the identified candidate predictors from the physical, psychological and social domains to develop two prediction models, followed by internal validation. These models will estimate the number of future days with NP and the severity of new NP episodes over 12 months.

## Methods

**Figure 1:**
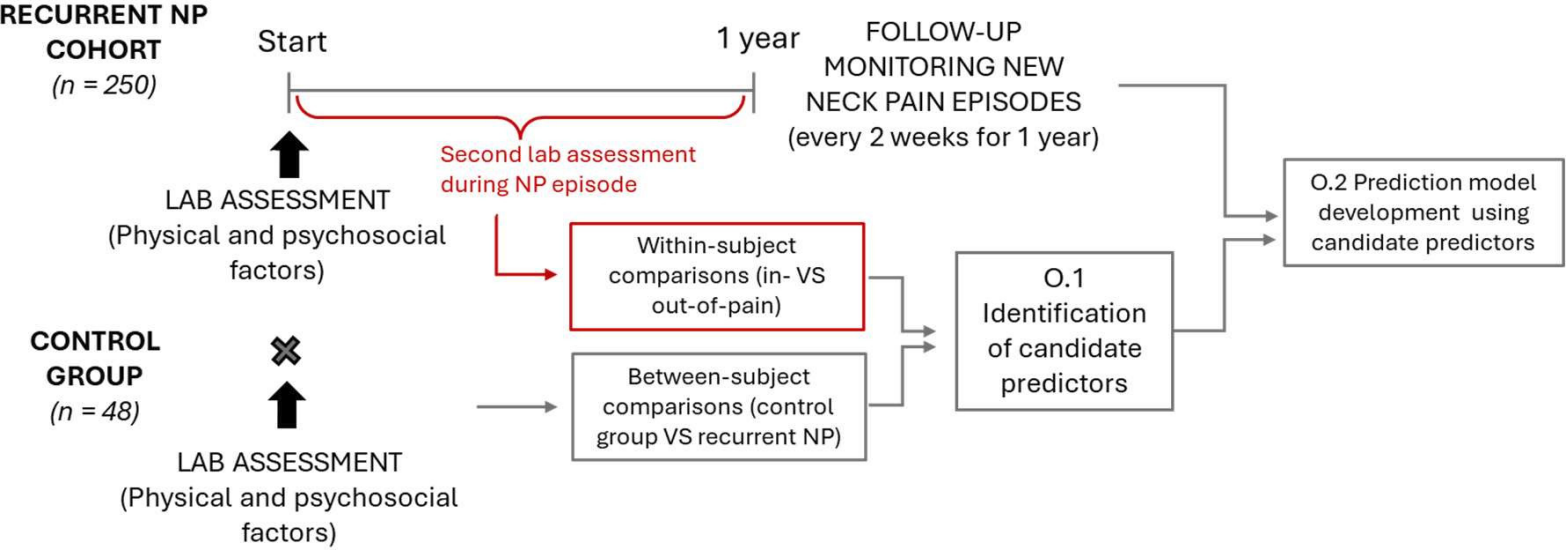
Study flow. NP, neck pain.

The END-RNP study is a multicentre prospective longitudinal cohort study; it is registered on the Central Portfolio Management System (CPMS ID: 69289) and receives support from the NIHR Research Delivery Network. This study will be conducted at the University of Birmingham, UK and University of Essex, UK from March 2025 to February 2028. The study will be reported according to the STROBE checklist and statement for transparent reporting of clinical prediction models that use regression or machine learning methods (TRIPOD+AI).^38 39^ A summary of the flow of the study is presented in figure 1. This study design has been informed by our Patient and Public Involvement and Engagement (PPIE) group. Specifically, the group provided feedback on the appropriate frequency and length of follow-up questionnaires, offered strategies to maximise participant engagement and helped identify potential predictors of spinal pain based on their lived experiences.

### Participants and recruitment

We will recruit male and female participants aged between 18 and 70 years. This upper limit is chosen to balance the inclusion of a wide adult age range against the confounding influence of advanced, age-related degenerative pathologies, whose prevalence and severity increase markedly in older populations.^40^ People reporting a history of recurrent episodes of NP but in remission (ie, pain-free) at the time of recruitment will be included in the recurrent NP cohort, and those without a history of NP will also be recruited as a control group to address the objective O.1. Inclusion criteria for the recurrent NP cohort will be the experience of two or more episodes of NP of idiopathic origin lasting more than 24 hours during the previous 12 months, separated by a period of remission, and with pain intensity greater than 2 on an 11-point numerical rating scale.^41^ People with recurrent NP tested during a period of remission need to be pain-free over the prior 30 days. The control group will include adults with no history of neck or shoulder pain that limited their daily function. Individuals will be excluded from both groups if they report chronic NP (ie, pain in the NP region from more than 3 months) or an acute episode of NP at time of recruitment, previous shoulder or spinal surgery, history of neck trauma, cervical radiculopathy/myelopathy, previous fracture in the neck or shoulder region, spinal deformity, neurological or cardiovascular disorders, rheumatic joint disease, possibility that they might be pregnant or any contraindications to physical exertion. Recruitment will be conducted between September 2025 and December 2026.

Participants with recurrent NP will be recruited from the community in Birmingham and in Essex. The recruitment process will aim to reflect the real-world distribution of NP occurrences between sexes. Given the 2:1 ratio of NP prevalence in favour of females,^42^ we expect that the recruitment process will result in a proportional sample that mirrors this difference. Due to the use of questionnaires and standardised procedures in the evaluation of candidate predictors and outcomes, only people able to understand or follow study procedures will be recruited. Participants for the control group will only be recruited from the community in Birmingham, and we will ensure comparable demographic characteristics to those within the recurrent NP cohort. Participants will be recruited from the University of Birmingham and University of Essex staff/student population and Birmingham’s/Essex’s local community. Recruitment methods will include printed hard copy advertisements with researchers’ emails pasted throughout the campus within the University of Birmingham and University of Essex, and identical soft copy advertisements disseminated using the University’s intranet and email system, social media platforms such as Facebook and *X,* and via word of mouth. Additionally, NIHR Research Delivery Network support will allow us to recruit participants with recurrent NP through the NIHR 'Be Part of Research’ service.

### Prognostic variables of pain recurrences

#### Baseline laboratory assessment

All recruited participants with a history of recurrent NP episodes will attend an initial laboratory session (in Birmingham or Essex) during a period of symptom remission, and a battery of tests will be conducted to obtain a comprehensive evaluation (table 1). To minimise inter-site variability, all assessments will follow a standardised protocol, and both laboratories will use identical equipment from the same manufacturer. Participants will also be asked to complete a series of questionnaires to assess psychological features, including the Depression, Anxiety and Stress Scales (DASS-21),^43^ the short version of the Tampa Scale for Kinesiophobia (TSK-11),^44^ the EQ-5D-5L^45^ and pain processing characteristics, including the pain-self efficacy questionnaire,^46^ pain coping inventory^47^ and central sensitisation inventory.^48^ Overall, we will collect information on demographic characteristics, psychological and social factors, pain processing, neck kinematics, neck sensorimotor control and neck muscle function.

**Table 1:** Collected data within physical, psychological and social domains

Domain	Feature extracted	Measurement tool
Demographics and Neck Pain characteristics	Age Sex BMI Ethnicity Number of other pain sites Number of previous pain episodes	Single question for each feature
Psychosocial factors	Stress (0–21) Anxiety (0–21) Depression (0–21) Kinesiophobia (11-44) Quality of life (0–100) Occupation Education level	DASS-21 DASS-21 DASS-21 TSK-11 EQ-5D-5L Single question Single question
Pain processing	Self-efficacy (0–60) Pain coping (12–48 active, 22–84 passive) Central sensitisation (0–100) Pressure pain threshold (kPa) Conditioned pain modulation	Pain Self-Efficacy Questionnaire Pain Coping Inventory Central sensitisation inventory Pressure algometry Cuff pressure algometry
Kinematic features and sensorimotor control	Average range of neck motion in flexion-extension, lateral flexion (R/L), rotation (R/L) Average speed of neck movement in flexion-extension, lateral flexion (R/L), rotation (R/L) Smoothness of neck movement (Jerk Index) in flexion-extension, lateral flexion (R/L), rotation (R/L) Neck proprioception (joint reposition error during right and left rotation)	All features obtained using wearable Inertial Measurement Unit (IMU, Sensamove 3D Cervical Trainer)
Neck muscle function and neuromuscular control	Craniocervical flexion performance Neck flexor strength and endurance Neck extensor strength and endurance Neck muscle co-contraction Neck muscle fatigue Upper trapezius strength	All features obtained using a hand-held dynamometer (NOD) and surface electromyography (Ultium EMG system)

BMI, body mass index; DASS-21, Depression, Anxiety, and Stress Scales; TSK-11, Tampa Scale for Kinesiophobia.

The same laboratory assessment will be conducted for the control group to identify potential candidate predictors (O.1), which have been selected for assessment based on findings from our previous work, current literature and PPIE input. The following physical tests will be performed in the same order as described below.

##### Pressure pain threshold

A pressure pain algometer (Somedic, Sweden) will be used to assess pressure pain threshold (PPT). PPT will be tested at the articular pillar of C2-C3 and C5-C6 facet joint, the upper trapezius muscle (midway between C7 and acromion), and over the tibialis anterior muscle (upper one-third of the muscle belly) over the dominant side.^49 50^ The measurement will be repeated three times, and the average will be taken for each site. This test has demonstrated high intrarater and interrater reliability in people with and without acute NP and can discriminate between those with and without NP.^50 51^

##### Conditioned pain modulation

Conditioned pain modulation (CPM) will be measured with cuff pressure algometry. An inflatable pressure cuff (NociTech, Denmark) will be mounted on the participant’s dominant arm. The pressure will be inflated, and the participant will indicate when they first detect pain (ie, pain detection threshold, PDT). The pressure will then continue to be increased until the subject’s tolerance limit, and this cuff pressure will be taken as the pressure tolerance threshold (PTT). Then the cuff will be immediately deflated. The same procedure will then be repeated on the non-dominant arm. To assess CPM, the cuff will be inflated to 70% of PTT on the non-dominant arm with PDT and PTT reassessed on the dominant arm. The CPM effect will be calculated as the conditioned PDT or PTT value minus the measures taken at baseline.^52^ User-independent cuff algometry has demonstrated high reliability for CPM assessment in people with chronic pain^53^ and has shown superior reliability compared with user-dependent assessments.^52^

##### Neck kinematics

A wearable Inertial Measurement Unit (IMU) system (myoMOTION Research Pro, Noraxon USA Inc.) with a sampling rate of 100 Hz will be used to analyse cervical kinematic outcomes while sitting. The IMU sensors will be placed via double-sided tape on the forehead and upper thoracic spine (T1). Participants will be asked to perform their active neck movements as far as they comfortably can go (flexion/extension, rotation, side flexion; randomised order using random number generator). Movements will be self-paced. They will be asked to do three repetitions for each plane of movement. One minute of rest will be provided between the different directions. The following features will be extracted: range of motion, speed of motion and smoothness of movement, measured by analysing the jerk index during active cervical movements.^54^ IMU sensors have high concurrent validity and reliability in the assessment of neck movements.^55^,^57^

##### Cervicocephalic relocation test

The same wearable IMU system (myoMOTION Research Pro, Noraxon USA Inc.) will also be used to test neck proprioception while sitting. Participants will be told to close their eyes and from a neutral starting position, subjects will perform active neck rotation and then they will return, as accurately as possible, to the initial position. The joint reposition error (absolute discrepancy between the initial and final position) will be analysed. Three repetitions will be performed for right and left rotation (random order). High reliability of IMU sensors and good-to-excellent agreement between IMU sensors and 3D motion capture have been demonstrated for the cervicocephalic relocation test.^58 59^

##### Craniocervical flexion test (CCFT)

Participants will perform the Craniocervical flexion test (CCFT) in a supine position. A handheld dynamometer will be used (NOD, OT Bioelectronica, Italy)^60^ to assess neck muscle strength, and surface electromyography (EMG) will be used to measure neck muscle activity. The handheld dynamometer, which will be used, is a device that has shown strong validity and high reliability for the assessment of neck muscle strength.^61 62^ Furthermore, the CCFT has high reliability, moderate convergent validity when compared with electromyography measures of neck muscles, and discriminative validity when testing people with and without NP.^63 64^ The NOD device will be placed behind the upper region of the neck. Two maximal isometric contractions (3–4 s) will be performed, and the force level will be recorded; 1 min rest will be provided between contractions. Next, participants will perform four contractions with different intensities: 20-40-60 and 80 per cent of the MVC of the CCFT. Each repetition will last 10 s, and 30 s of rest will be allowed between stages. During this test, visual feedback of the force output will be provided to participants. They will then perform a single sustained contraction at 40% MVC for 20 s. EMG signals will be recorded from the sternocleidomastoid muscle bilaterally using surface electrodes with a fixed 2 cm interelectrode distance.^65^ Surface electromyography will be measured with the Ultium EMG System (Noraxon, USA) using wireless sensors. The sampling frequency of EMG signals is 2000 Hz with the data converted to digital form by a 16-bit A/D converter, and a 10–500 Hz bandwidth filter is applied by the amplifier. Signals will be recorded and visually checked using the software myoRESEARCH 4 (Noraxon, USA).

##### Maximal and submaximal voluntary contractions

The same handheld dynamometer and surface electromyography system will be used to measure neck muscle activity during maximum and submaximal neck flexion and extension contractions. The activity of the sternocleidomastoid and splenius capitis muscles will be detected using surface electrodes with a fixed 2 cm interelectrode distance and placed bilaterally following guidelines for electrode placement.^65^,^67^ Flexion and extension isometric forces of the neck muscles will be assessed with the participants seated with their back supported and their torso firmly strapped to the seat back. Participants will be instructed to perform maximal neck flexion and extension against the dynamometer held by the assessor at the forehead and occiput respectively. Electromyographic measures of the superficial neck muscles have been shown to be reproducible and can discriminate between individuals with and without NP.^32 68 69^

Maximal isometric contraction while seated: following a period of familiarisation with the device, participants will be asked to perform two maximal contractions for both flexion and extension, which will be done in a random order. Each contraction will last 3–4 s, with 1 min rest between each repetition. The highest value for each direction will be considered as the reference maximum voluntary contraction (MVC) in order to calculate the submaximal force value. EMG signals will be recorded.

Submaximal contraction while seated: after 5 min of rest, participants will perform one submaximal isometric contraction in flexion and one in extension (order randomised for each participant) at 40% of MVC for 20 s. 5 minutes of rest will be provided between contractions. EMG signals will be recorded. Participants will be asked to rate their exertion using the Borg CR10 Scale (0–10) immediately following the completion of each sustained submaximal contraction.

Finally, participants will be asked to perform a shoulder shrug to measure the maximal voluntary contraction of the upper trapezius. The dynamometer will be placed over the participant’s acromion region by the investigator, who will resist the force exerted by the participant while they shrug their shoulder. Participants will be asked to perform two maximal contractions for each side, and the order will be randomised.

##### Neck flexor and extensor endurance

Neck flexion endurance will be measured with the patient in supine. The participant will slightly nod and then raise the head just above the examination table. For the test of neck extension endurance in prone, the head will be placed away from the examination table so that an inclinometer will be attached to the head to indicate 0 degrees when in neutral. Additionally, a 3-kg weighted strap will be secured around the head. The participant will hold the position until they have a change in head angle of five or more degrees for extension. Endurance time will be recorded for each test. A rest of 10 min will be given between the tests, and the tests will be conducted in a random order. A familiarisation of the movements will be given. Good to excellent reliability and validity, assessed by comparison with the Neck Disability Index, have been demonstrated for isometric muscle endurance tests in people with NP.^70 71^

### Second laboratory assessment during a NP episode

People in the recurrent NP cohort who develop a new painful NP episode after the baseline assessment and who agree to participate in a further laboratory session will return to the lab and the same tests and questionnaires performed during the baseline assessment will be conducted to evaluate changes in physical and psychological features during a painful episode (table 1). This will aid in identifying potential candidate predictors (O.1) that are influenced by pain and clinically relevant. Although such changes during a NP episode may represent a direct consequence of pain, this does not preclude their prognostic relevance. Features that are affected by pain and not fully restored during remission may persist or progressively worsen over time, potentially contributing to the development of future NP episodes.

### Outcomes of interest

The development of new NP episodes and their severity represent the main outcomes of interest in the recurrent NP cohort. To define the presence and severity of NP episodes, multiple questions will be collected through online questionnaires and by referring to the previous 2 weeks recall period (table 2). The collected information will be used to identify the number of days with NP during the 12-month follow-up. Furthermore, average and worst pain intensity (rated using a visual analogue scale 0–100) during new NP episodes over the 12 months will be aggregated to define the severity of NP. Based on established cut-points for grading chronic musculoskeletal pain,^72^ participants will then be classified into four severity groups: No pain (score of 0), mild pain (score of 1–34), moderate pain (score of 35–64) and severe pain (score >64). These two continuous and ordinal outcomes (ie, days with NP and severity of NP) will be the dependent variables used to develop the prediction models. Although we recognise that some participants might develop chronic pain during the follow-up, we decided to specifically focus on the frequency and severity of new NP episodes to better fit the fluctuating nature and symptom trajectory of people with recurrent NP.

**Table 2:** Collected data on neck pain recurrences

Domain	Features extracted	Measurement tool
Recurrent episodes	Number of days with NP Average and worst NP intensity (VAS, 0–100) NP interference in daily activities (VAS, 0–100) NP interference in recreational, social and family activities (VAS, 0–100) NP interference with work (VAS, 0–100) Number of days using medication for NP relief Number and type of treatment received	Single questions

NP, neck pain; VAS, visual analog scale.

#### Follow-up assessment of new NP episodes and other factors

During the 12-month follow-up period, we will monitor the presence of new NP episodes. Specifically, people recruited in the recurrent NP cohort will receive an email with a link to an online questionnaire every 2 weeks throughout the entire 12-month follow-up period, commencing 2 weeks after the baseline assessment. This questionnaire seeks information on new NP episodes (table 2), and it will be sent automatically using the REDCap system, which is a secure, web-based application designed to support data capture for research studies. Reminders will be sent via email every 24 hours for a maximum of three consecutive days. A frequency of 2 weeks has been chosen to reduce the risk of recall bias and is informed by our PPIE group, which indicated that 2 weeks represents a tolerable amount of time for follow-up questionnaires over 12 months. In addition to information on new NP episodes, online follow-ups will also seek information on other environmental, behavioural and psychosocial factors including perceived stress, fatigue, quality of life, social support, level of physical activity, average sleep hours and quality and work-related factors. These factors will be collected to explore how new NP episodes interfere with individuals’ lives.

### Data analysis plan

#### O.1 Identification of candidate predictors

To identify candidate predictors that are clinically relevant, we will conduct two different comparisons. First, we will assess at baseline physical, psychological and social features during a period of remission and compare them with normative values from a control group using an independent t-test if the data satisfy parametric assumptions, or a non-parametric test, such as the Mann-Whitney U test, if they do not. Additionally, collected features within the physical and psychological domains will be assessed during a pain episode to evaluate the impact of pain on them. Comparisons with baseline (without pain) will be conducted using a paired t-test or a non-parametric alternative such as the Wilcoxon signed-rank test for within-subject comparisons. Differences will also be quantified by mean differences and 95% CIs. Physical, psychological and social features that differ between groups or within the same subject (during remission and pain episode) will then be considered as candidate predictors. Importantly, only baseline values of the candidate predictors collected will be used for prediction model development.

#### O.2 Development and internal validation of prediction models

We will use linear and ordinal regression models (with either a Least Absolute Shrinkage and Selection Operator (LASSO) penalty or a uniform shrinkage factor to account for any overfitting) to develop models for the prediction of continuous and ordinal outcomes, respectively. LASSO allows variable selection while incorporating shrinkage to deal with overfitting^73^, whereas the uniform shrinkage factor is estimated post-model fitting using bootstrapping and then applied to all predictor effects. The stability of performance of each development approach will be examined and compared. To ensure more stable models, the number of candidate predictors will be determined by the total sample size (number of participants and events) based on Riley *et al*^74^ (see sample size section). Candidate predictors will include those identified in O.1, and continuous predictors will be handled on their continuous scale allowing for potential non-linearity with fractional polynomials. Repeated cross-validation will be used to estimate tuning parameters (penalty terms) in the LASSO. Bootstrapping will be used for internal validation (repeating all model development steps, including any from O.1 to identify candidate predictors) to obtain a uniform shrinkage factor and to examine the stability of the model’s predictions and the potential for miscalibration in new data,^75^ and to produce optimism-adjusted measures of performance including R^2^, C-statistic (for ordinal outcome model), calibration-in-the-large and calibration slope, and prediction and classification instability plots and indices.^75^ Calibration plots with calibration curves will also be presented. The models’ predictive performance will be examined across different ethnic groups and by region, where possible, as part of fairness checks. For the ordinal outcome model, clinical utility will be examined using net benefit and decision curves, with a range of risk thresholds identified through consultations with patients and clinicians.^76^ Missing data in the candidate predictors is anticipated to be minimal, but if necessary, it will be managed using multiple imputation by chained equations method, which assumes the data are Missing at Random (MAR). Results will be averaged across imputation datasets to produce the final model and performance statistics. Multiple imputation at development is congenial with having no missing predictor data at deployment, which is as anticipated. For the ordinal outcome model, if the number of outcome events is sparse in some categories, we will collapse with neighbouring categories to address this issue. Specifically, we anticipate it may be necessary to collapse the no NP and mild NP categories, and the moderate NP and severe NP categories.

### Sample size

#### Sample size estimation for identification of candidate predictors (O.1)

Potential candidate predictors will be identified after testing physical and psychosocial factors for between-subject and within-subject differences. To consider a feature as a potential predictor, we expect a moderate effect size both for the comparison between subjects and within subjects. For the between-subjects analyses, we will consider 48 participants for the control group and 96 for the recurrent NP group. The sample size has been determined through a power analysis (G*Power), which assumes a moderate effect size of 0.5, an alpha level of 0.05 and a beta level of 0.8, with an allocation ratio of 2:1. For the within-subject comparison, we will conduct the second lab assessment on 34 participants with recurrent NP during an acute episode (α=0.05 and β=0.8).

#### Sample size estimation for the development and internal validation of prediction models (O.2)

To develop a prediction model for the continuous outcome of days with NP over the following 12 month (continuous outcome), we aim to recruit 250 participants with recurrent NP. The desired sample size is selected to target a shrinkage factor of ≥0.9 and small optimism in R² (≤ 0.05) to minimise overfitting.^74^ The calculation assumes an R² value of 0.40 based on previous data reported in a systematic review of prognostic models for neck pain,^77^ a mean of 35 days with NP and a residual SD of 15. The calculation suggests that about 16 candidate predictors can be considered with 250 participants. The sample size is also large enough to estimate the overall mean, which preliminary data suggests is about 35 days with NP over a 12-month period. Accounting for a drop-out rate at 20% due to the long follow-up, we aim to recruit 300 participants with recurrent NP over two testing sites (Birmingham and Essex). Uncertainty of predictions will be quantified using sampling distributions derived from bootstrapping.

For the severity of NP recurrences (ordinal outcome), we assume a predicted outcome distribution of 10% (no NP), 40% (mild NP), 40% (moderate NP) and 10% (severe NP), which was informed by preliminary data. Using a sample size of 250 participants (as above) allows 10 predictor parameters to minimise model overfitting assuming a Nagelkerke R-squared of 0.4 and targeting a 95% CI width of 0.12 for the overall risk,^74^ for a logistic regression model after collapsing the outcome to two categories as outlined above. We expect the ordinal regression model to allow more than 10 parameters, and we will consider 16 to be consistent with the continuous outcome and examine the impact of this using prediction and classification instability plots.^75^

### Patient and public involvement

The development of this study was informed by discussion with our PPIE group. A PPIE member will be included in the programme-management group for the full duration of the project. Regular meetings with the PPIE group will be held throughout the study to gather feedback on progress, maximise participant recruitment and engagement, and support dissemination of the findings. PPIE involvement will be described using the GRIPP2-SF alongside the results.^78^

## Discussion

The findings from this project will reveal factors that can help predict the development of future NP episodes, providing clinically meaningful insights and supporting the development of a practical prediction tool to assist clinicians in identifying patients at higher risk of experiencing new and more severe NP episodes. By investigating several modifiable variables within a biopsychosocial framework as potential prognostic factors, the identified predictors of future NP episodes could represent potential treatment targets to be investigated in future research. A limitation of the proposed design is that assessments are conducted within a laboratory setting, and the generalisability of the findings to routine clinical practice and their feasibility in standard physiotherapy settings will require further investigation. However, clinical translation is not intended to rely on direct adoption of the full laboratory test battery. Rather, the identification of a reduced set of clinically meaningful predictors represents a key step toward translation, particularly given that several of the physical assessments rely on technologies that are increasingly accessible to clinicians, or can be conducted with simplified devices with demonstrated reliability and validity, such as smartphones for neck kinematic assessment or app-integrated sensor features.

### Ethics and dissemination

The END-RNP study was approved by the Ethics Committee at the University of Birmingham (ERN_4005-Aug2025) and by the University of Essex (ETH2526-0098) on the 2 September 2025, prior to the recruitment of the first participant. All participants will provide informed electronic consent before taking part in the study.

The findings will be presented at national and international conferences and submitted for publication in peer-reviewed journals. We plan to publish our de-identified data in a public, open-access repository within 1 year after the completion of the project. We also plan to publish the same data on the Alleviate data hub (https://alleviate.ac.uk). Based on the stability of the prediction model, we will develop a nomogram intuitive for clinicians to use. This tool will facilitate further evaluation of the model (e.g. external validation studies) and ongoing management of people with NP by providing predictive insights into patient risk for future NP episodes. We will present at CPD meetings organised by Birmingham Community Healthcare for stakeholder involvement with clinical staff and for dissemination of findings. We will further engage with clinicians via a planned workshop in the final year of funding to present the findings of the project and initiate a discussion on the translation of these findings to clinical practice.
