# Supplementary material for: Multidomain evaluation and data-driven approaches to predict recurrent neck pain (END-RNP): a study protocol for a multicentre prospective longitudinal cohort study
Source: BMJ Open. 2026 Feb 27;16(2):e112430. doi: 10.1136/bmjopen-2025-112430 (PMC12959022; doi:10.1136/bmjopen-2025-112430)
Supplement: Supplementary file 1 [file bmjopen-2025-112430.reviewer_comments.pdf]

PEER REVIEW HISTORY

BMJ Open publishes all reviews undertaken for accepted manuscripts. Reviewers are asked to complete a checklist review form and are provided with free text boxes to elaborate on their assessment. These free text comments are reproduced below.

ARTICLE DETAILS

Title (Provisional)

Multidomain Evaluation and Data-Driven Approaches to Predict Recurrent Neck Pain (END-RNP): A study protocol for a multicentre prospective longitudinal cohort study

Authors

Devecchi, Valter; Liew, Bernard; Price, Jonathan; Snell, Kym; Riley, Richard; Falla, Deborah

---

VERSION 1 - REVIEW

**Reviewer**	**1**
**Name**	**Verwoerd, Martine**
**Affiliation**	**Utrecht University**
**Date**	**22-Dec-2025**
**COI**	**None**

Thank you for the opportunity to review this manuscript. Overall, this is a well-written and methodologically ambitious study. I appreciate the authors' effort to address recurrent neck pain, which is a clinically relevant yet comparatively under-researched subgroup. The manuscript is clear, the laboratory procedures are described in detail, and the study design appears comprehensive.

At the same time, I have several points that, if addressed, could strengthen the rationale and improve the interpretability and clinical relevance of the work.

Major comments

Clarify definitions and target populations (chronic vs recurrent)

The manuscript would benefit from explicit operational definitions of recurrent and chronic neck pain (e.g., duration, pain-free periods, number of episodes, minimum remission length). At present, it is not fully clear which subgroup the study primarily aims to represent and how participants are classified.

Relatedly, it may be useful to briefly position recurrent neck pain relative to chronic neck pain without pain-free periods. If the focus is recurrent pain, please clarify why chronic pain

is not included, or, if both are included, how differences between these groups are handled analytically and conceptually.

Prognostic relevance of physical impairments: rationale and distinction from-cross sectional differences

A key conceptual issue is the distinction between (a) physical impairments observed during a pain episode and (b) prognostic factors that predict recovery, recurrence, or chronification. Physical differences observed in a chronic pain stage do not necessarily imply prognostic value.

In this context, it may be helpful to more explicitly justify why the selected physical domains are expected to have prognostic value in recurrent neck pain.

Validity and reliability of laboratory tests

The laboratory assessments are described clearly and seem reproducible. However, I did not find sufficient information on the measurement properties of the selected tests, particularly those assessing neck muscle function and neuromuscular control.

Please clarify:

- Which measures have established validity and reliability in the relevant population and setting?

- For measures with limited psychometric evidence, what is the rationale for their inclusion as candidate predictors, and how will measurement error be addressed?

- Are there references supporting test–retest reliability, inter-rater reliability, and/or responsiveness for the outcomes used?

Given that the aim is prognostic modelling, the measurement properties of candidate predictors are critical for interpreting associations and for translation.

Role of pain during testing and its implications

The potential influence of pain on physical test performance is an important consideration (and it is good that the authors include pain responses). However, the manuscript would benefit from a clearer rationale explaining how and why pain at the time of testing may affect performance outcomes, and how this is handled in the interpretation.

Relatedly, if measures differ substantially between remission and pain episodes, this may partly reflect state effects (pain-related performance changes) rather than stable risk markers. Explicitly discussing this distinction would strengthen the conceptual framework.

Selection of candidate predictors based on within-person change: justification needed

I did not fully understand the rationale for restricting candidate predictors to those that show within-subject differences between remission and a pain episode. A variable that does not change across these time points could still be prognostic (e.g., a stable vulnerability

factor). Conversely, a variable that changes may primarily be pain-dependent rather than predictive of future outcomes.

Please provide a clear justification for this selection strategy. More generally, selecting candidate predictors purely based on Aim 1 could be problematic for the broader goal of prognostic modelling (i.e., optimising prediction). If sample size allows, I would encourage the authors to consider including a broader set of theoretically relevant predictors in the modelling phase, rather than excluding variables solely because they do not show within-person change.

A similar point applies to psychological variables: for example, depressive symptoms may vary as a consequence of pain rather than precede it. If the intent is to identify prognostic predictors, it would be valuable to clarify whether variables are expected to precede episodes (risk markers) versus co-occur (state markers), and how this informs inclusion in prediction models.

Balance between cross-sectional and longitudinal findings

Several cited studies appear cross-sectional. Longitudinal evidence for physical factors is often drawn from whiplash populations, which may not generalise to non-traumatic neck pain. It would strengthen the introduction to more clearly distinguish traumatic versus non-traumatic neck pain evidence, and to state why extrapolation is justified (or where uncertainty remains).

Clinical translation and feasibility

Finally, I am uncertain how the proposed laboratory-based predictors can be implemented in routine clinical practice. If these assessments are not feasible in standard physiotherapy settings, it would be helpful for the authors to discuss:

• the intended pathway to translation (e.g., simplified clinical proxies, reduced test battery, tiered assessment),

• the practical value of the prognostic model if key predictors require specialised equipment or laboratory conditions.

Minor comments

The statement that "Previous research has often focused on a single domain, largely overlooking the variation in individual and contextual physiological responses to pain" may be somewhat overstated. Consider rephrasing to reflect the existing literature better.

VERSION 1 - AUTHOR RESPONSE

Reviewer: 1

Thank you for the opportunity to review this manuscript. Overall, this is a well-written and methodologically ambitious study. I appreciate the authors' effort to address recurrent neck

pain, which is a clinically relevant yet comparatively under-researched subgroup. The manuscript is clear, the laboratory procedures are described in detail, and the study design appears comprehensive.
At the same time, I have several points that, if addressed, could strengthen the rationale and improve the interpretability and clinical relevance of the work.

We thank the reviewer for their evaluation of the manuscript and for their positive feedback. We have addressed the important points raised and have revised the manuscript accordingly to strengthen the rationale and improve the interpretability and clinical relevance of the work.

Major comments

Clarify definitions and target populations (chronic vs recurrent)
The manuscript would benefit from explicit operational definitions of recurrent and chronic neck pain (e.g., duration, pain-free periods, number of episodes, minimum remission length). At present, it is not fully clear which subgroup the study primarily aims to represent and how participants are classified.
Relatedly, it may be useful to briefly position recurrent neck pain relative to chronic neck pain without pain-free periods. If the focus is recurrent pain, please clarify why chronic pain is not included, or, if both are included, how differences between these groups are handled analytically and conceptually.

We appreciate the reviewer's request to clarify the definitions and the importance of selecting the target populations. To identify the population of interest we aligned with the current definition indicating as criteria the "*experience of two or more episodes of NP of idiopathic origin lasting more than 24 hours during the previous 12 months, separated by a period of remission, and with pain intensity greater than 2 on an 11-point numerical rating scale.*" Furthermore, we specified that participants tested during a period of remission need to be pain-free for the prior 30 days. We also explicitly stated that individuals presenting with chronic neck pain (continuous pain without remission) or acute neck pain at the time of recruitment are excluded from the study.

We have expanded the Introduction to conceptually position recurrent neck pain relative to chronic pain. The text now reads: "*However, a limitation of previous studies was the lack of a clear distinction between recurrent and chronic NP. While chronic NP involves persistent symptoms, recurrent NP is characterised by distinct episodes of pain separated by symptom-free remission periods. This makes it challenging to distinguish between the two if outcomes are evaluated at a single time point, overlooking the different symptom trajectories of recurrent versus chronic NP*".

Regarding the outcome measure, we also specified that "*although we recognise that some participants might develop chronic pain during the follow-up, we decided to specifically focus on the frequency and severity of new NP episodes to better fit the fluctuating nature and symptom trajectory of people with recurrent NP.*"

Prognostic relevance of physical impairments: rationale and distinction from-cross sectional differences
A key conceptual issue is the distinction between (a) physical impairments observed during a pain episode and (b) prognostic factors that predict recovery, recurrence, or chronification. Physical differences observed in a chronic pain stage do not necessarily imply prognostic value.

In this context, it may be helpful to more explicitly justify why the selected physical domains are expected to have prognostic value in recurrent neck pain.

We thank the reviewer for highlighting the important distinction between cross-sectional physical impairments and factors with true prognostic relevance. We agree that physical differences identified during a pain episode do not necessarily imply predictive value for recovery or recurrence.

Our rationale for assessing physical domains is based on evidence that, in individuals with recurrent neck pain, resolution of pain does not necessarily coincide with recovery of physical function.[1] Physical impairments can persist during pain-free remission periods and therefore represent potential predisposing factors of future neck pain episodes, in accordance with current theories on motor adaptations to pain.[2,3]

We expanded the introduction with a better explanation of our rationale that "*in individuals with recurrent NP, the resolution of pain symptoms does not necessarily correspond to a recovery of physical function; sensorimotor impairments similar to those observed in chronic NP often persist during symptom remission [17], potentially acting as a predisposing factor for future NP episodes.*"

Validity and reliability of laboratory tests
The laboratory assessments are described clearly and seem reproducible. However, I did not find sufficient information on the measurement properties of the selected tests, particularly those assessing neck muscle function and neuromuscular control.
Please clarify:
- Which measures have established validity and reliability in the relevant population and setting?
- For measures with limited psychometric evidence, what is the rationale for their inclusion as candidate predictors, and how will measurement error be addressed?
- Are there references supporting test–retest reliability, inter-rater reliability, and/or responsiveness for the outcomes used?
Given that the aim is prognostic modelling, the measurement properties of candidate predictors are critical for interpreting associations and for translation.

We agree on the importance of highlighting the psychometric properties of the investigated tests, and we have added more information on the reliability and validity of each investigated assessment.

Role of pain during testing and its implications
The potential influence of pain on physical test performance is an important consideration (and it is good that the authors include pain responses). However, the manuscript would benefit from a clearer rationale explaining how and why pain at the time of testing may affect performance outcomes, and how this is handled in the interpretation.
Relatedly, if measures differ substantially between remission and pain episodes, this may partly reflect state effects (pain-related performance changes) rather than stable risk markers. Explicitly discussing this distinction would strengthen the conceptual framework.

We appreciate the reviewer feedback regarding the influence of pain on physical performance. Evidence from clinical and experimental pain studies, as well as systematic reviews, indicates

that neck pain alters physical function.[4,5] In addition, psychological factors such as fear of movement may further affect task execution.[3] Testing individuals while in pain, therefore, introduces considerable heterogeneity, which may compromise the generalisability of a prediction model and represents an important source of confounding. We expanded the introduction to clarify this point, and it now reads "*Additionally, previous studies have typically assessed participants at baseline while in pain. Testing during an acute painful episode can introduce significant heterogeneity, as the presence of pain alters physical function and psychological states [10,31,32], potentially masking the role of relevant prognostic factors. Therefore, recruiting and testing individuals with recurrent NP during a pain-free remission period provides a more homogeneous sample. This allows for the identification of predictors of recurrence without the confounding influence of current pain*."

Also, we acknowledge that measures differing between pain-free and painful states may partly reflect state-dependent effects rather than stable prognostic factors. However, these mechanisms are not necessarily mutually exclusive. If pain induces changes in physical function (e.g., reduced strength or altered motor control) and these changes are not fully restored during remission, such impairments may persist and plausibly contribute to an increased risk of subsequent neck pain episodes. We explained this rationale in the Methods section under "Second laboratory assessment during a NP episode" that now reads "*Although such changes during a NP episode may represent a direct consequence of pain, this does not preclude their prognostic relevance. Features that are affected by pain and not fully restored during remission may persist or progressively worsen over time, potentially contributing to the development of future NP episodes*."

Selection of candidate predictors based on within-person change: justification needed
I did not fully understand the rationale for restricting candidate predictors to those that show within-subject differences between remission and a pain episode. A variable that does not change across these time points could still be prognostic (e.g., a stable vulnerability factor). Conversely, a variable that changes may primarily be pain-dependent rather than predictive of future outcomes.
Please provide a clear justification for this selection strategy. More generally, selecting candidate predictors purely based on Aim 1 could be problematic for the broader goal of prognostic modelling (i.e., optimising prediction). If sample size allows, I would encourage the authors to consider including a broader set of theoretically relevant predictors in the modelling phase, rather than excluding variables solely because they do not show within-person change. A similar point applies to psychological variables: for example, depressive symptoms may vary as a consequence of pain rather than precede it. If the intent is to identify prognostic predictors, it would be valuable to clarify whether variables are expected to precede episodes (risk markers) versus co-occur (state markers), and how this informs inclusion in prediction models.

We thank the reviewer for this suggestion and agree that candidate predictors should not be limited to variables showing within-subject differences between remission and pain episodes. Accordingly, in addition to within-person changes, we will also consider variables that differ between individuals with a history of recurrent neck pain tested while in symptom remission at baseline and a control group without a history of neck pain.

Moreover, we will include variables with established prognostic relevance for recurrent or persistent neck pain based on recent systematic reviews [28,29]. These points have been clarified in the Introduction in relation to Objective 1.

Balance between cross-sectional and longitudinal findings
Several cited studies appear cross-sectional. Longitudinal evidence for physical factors is often drawn from whiplash populations, which may not generalise to non-traumatic neck pain. It would strengthen the introduction to more clearly distinguish traumatic versus non-traumatic neck pain evidence, and to state why extrapolation is justified (or where uncertainty remains).

We agree that findings from whiplash populations need to be interpreted cautiously when extrapolated to individuals with idiopathic neck pain. Accordingly, we have clarified this distinction in the Introduction by stating that "*whiplash-associated disorders and idiopathic NP differ in aetiology and have been shown to present distinct physical and sensorimotor profiles [34-36]. As such, findings from people with whiplash-associated disorders may not fully generalise to non-traumatic neck pain, highlighting the need to directly investigate prognostic physical factors in individuals with recurrent idiopathic NP*." We have also clarified in the study aims that the present study specifically focuses on individuals with idiopathic neck pain.

Clinical translation and feasibility
Finally, I am uncertain how the proposed laboratory-based predictors can be implemented in routine clinical practice. If these assessments are not feasible in standard physiotherapy settings, it would be helpful for the authors to discuss:

- the intended pathway to translation (e.g., simplified clinical proxies, reduced test battery, tiered assessment),
- the practical value of the prognostic model if key predictors require specialised equipment or laboratory conditions.

Although the protocol includes an extensive laboratory-based assessment, our intention is to obtain a parsimonious prognostic model that is accessible and clinically applicable. The proposed pathway to translation is not the direct implementation of the full test battery, but rather the identification of a reduced set of clinically meaningful predictors.

Importantly, many of the physical assessments used in this study rely on equipment that is already available or increasingly accessible in clinical settings. For example, kinematic variables can be captured using a single inertial measurement unit or smartphone-based sensors, for which previous systematic reviews have demonstrated good reliability and validity. Similarly, handheld dynamometry is widely used in clinical practice.

We have clarified this point in the Discussion, where we acknowledge that laboratory-based assessment represents a limitation and explicitly discuss the feasibility of translation through reduced test batteries and simplified technologies (e.g., smartphone-based kinematic assessment or app-integrated sensor features).

Minor comments
The statement that "Previous research has often focused on a single domain, largely overlooking the variation in individual and contextual physiological responses to pain" may be somewhat overstated. Consider rephrasing to reflect the existing literature better.

We agree on the suggestion, and we revised the sentence that now reads "*Although previous research has examined multiple domains within a biopsychosocial framework, individual and contextual physiological responses to pain have received less attention [27].*"

REFERENCES:

1. Devecchi V, Rushton AB, Gallina A, Heneghan NR, Falla D. Are neuromuscular adaptations present in people with recurrent spinal pain during a period of remission? a systematic review. PLoS One. 2021 Apr 1;16(4):e0249220. doi: 10.1371/journal.pone.0249220.
2. Hodges PW, Tucker K. Moving differently in pain: A new theory to explain the adaptation to pain. Pain. 2011;152:S90–8. doi: 10.1016/j.pain.2010.10.020
3. Vlaeyen JWS, Crombez G. Behavioral Conceptualization and Treatment of Chronic Pain. Annu Rev Clin Psychol. 2020;16:187–212. doi: 10.1146/annurev-clinpsy-050718-095744
4. Dagli AC, Yazgan Dagli B, Al-Yahya E, Fonseca L, Caleb-Solly P. The evidence for biomechanical and physiological parameters as biomarkers to discriminate between individuals with and without non-specific neck pain using sensor devices: A systematic review with meta-analysis. J Pain. 2025 Nov;36:105543. doi: 10.1016/j.jpain.2025.105543.
5. Cabral HV, Oxendale C, Devecchi V, Falla D, Gallina A. The Effect of Experimentally Induced Pain in the Cervical, Shoulder, or Orofacial Regions on Cervical Neuromuscular and Kinematic Features: A Systematic Review and Meta-analysis. J Pain. 2024 Dec;25(12):104660. doi: 10.1016/j.jpain.2024.104660.
